# The medical decision-making process in the time of the coronavirus pandemic

**DOI:** 10.5935/0103-507X.20200033

**Published:** 2020

**Authors:** Cassiano Teixeira, Regis Goulart Rosa, Edison Moraes Rodrigues Filho, Eduardo de Oliveira Fernandes

**Affiliations:** 1 Unidade de Terapia Intensiva, Hospital de Clínicas de Porto Alegre, Universidade Federal do Rio Grande do Sul - Porto Alegre (RS), Brasil.; 2 Unidade de Terapia Intensiva, Hospital Moinhos de Vento - Porto Alegre (RS), Brasil.; 3 Unidade de Clínica Médica, Hospital Nossa Senhora da Conceição - Porto Alegre (RS), Brasil.

**Keywords:** Clinical decision-making, Metacognition, Feedback, Pandemics, Coronavirus infectious, Coronavirus, Betacoronavirus, Catastrophic illness, Intensive care units, Tomada de decisão clínica, Metacognição, Retroalimentação, Pandemias, Infecções por coronavírus, Coronavirus, Betacoronavirus, Doenças catastróficas, Unidades de terapia intensiva

## Abstract

The disease pandemic caused by the novel coronavirus has triggered significant changes in the medical decision-making process relating to critically ill patients. Admissions to intensive care units have suddenly increased, but many of these patients do not present with clinical manifestations related to the viral infection but rather exacerbation of preexisting diseases. In this context, we must prevent intuitive decision-making and insecurity from leading us to exhaust the available critical-care beds before they are truly necessary, while still recognizing the importance of rapid decision-making in emergency situations. One of the best ways to achieve this goal may be by practicing metacognition and establishing ways for regular feedback to be provided to professionals engaged in inherently rapid decision-making processes.

## Abstract (pt)

A pandemia causada pelo novo coronavírus tem provocado mudanças significativas no processo de tomada de decisão médica diante do paciente grave. Repentinamente, aumentaram as admissões em unidades de tratamento intensivo, porém, muitos desses casos não apresentam quadros relacionados à infecção viral, mas à exacerbação de doenças preexistentes. Nesse contexto, precisamos evitar que o processo decisório intuitivo e a insegurança nos levem a exaurir a disponibilidade de leitos críticos, antes do momento em que eles sejam realmente necessários, mesmo reconhecendo a importância do método decisório rápido em situações emergências. Uma das melhores formas de atingir esse propósito talvez seja por meio da prática da metacognição e da estruturação de formas de *feedback* regulares aos profissionais envolvidos em processos decisórios inerentemente rápidos.

## INTRODUCTION

The disease pandemic caused by the novel coronavirus has triggered significant changes in the medical decision-making process relating to critically ill patients. Admissions to the intensive care unit (ICU) of patients suspected to be infected with the severe acute respiratory coronavirus 2 (SARS-CoV-2) have suddenly increased. Such patients fit into two phenotypes that require distinct management and surveillance levels: patients with exacerbations of preexisting underlying diseases and patients with severe forms of viral pneumonia (respiratory failure, for example). These two patient groups may currently be managed in similar ways, neglecting their specific needs - a factor that may contribute to the increased risk of treatment failure, iatrogenesis, and inequities in access to intensive care and resource allocation. What has happened in the medical decision-making process? It is not reasonable to believe there has been a sudden reduction in declarative knowledge, as the signs and symptoms of the main indications for intensive care have not changed. However, the organization of technical knowledge for generating appropriate diagnoses and management actions - procedural knowledge - indeed seems to be at risk of impairment, considering the multiple and rapid pressures generated by the pandemic we are experiencing. This complex behavior may be explained by cognitive biases related to the novel coronavirus pandemic. Cognitive bias (or cognitive tendency) is characterized by a pattern of distorted judgment that occurs in particular situations, leading to perceptual distortion, inaccurate judgment, illogical interpretation, or what is widely called “irrationality.”^(1)^

In essence, the diagnostic process aims to determine what disease the patient has based on a set of signs and symptoms.^(2-4)^ More experienced doctors use intuitive and automatic cognitive processing - the infamous “clinical eye.” Basically, this system relies on heuristics (mental shortcuts) to assess to what extent the patient’s symptoms fit the patterns and prototypes of diseases that professionals have stored in their memory. Of course, this form of processing requires extensive experience with clinical cases and previous experience with many diseases.^(2,3)^ Younger professionals mainly use a more reflective, analytical, and rational form of information processing that involves the application of the hypothetico-deductive method for each case. Based on the observations of a particular case, the doctor generates hypotheses about possible alternative diagnoses that may fit the symptoms and, from there, through exams, tests, and analyses, discards less probable hypotheses until arriving at a correct diagnosis.^(2,4)^ This latter process is a highly systematic procedure that consumes many cognitive resources, in addition to being more time-consuming than the former process.^(2,3)^ Apparently, doctors commonly use both systems together, which makes the overall process more reliable and safe for diagnostic decision-making.

The rapid and intuitive process is not necessarily irrational, although it is basically nonconscious.^(5)^ Although not consciously analytical, it can involve extensive processing of previously learned information and can provide, as a result, a feeling about the best decision to be made, often through autonomic signs or symptoms - Damasio’s somatic markers.^(6)^ The rapid process is fundamental in situations involving many variables and urgency of time and resources. This is because the human brain has difficulty consciously analyzing situations with several involved variables.^(7)^ In the decision-making process, both faster and slower processes are fundamental.^(8)^ In emergency situations, intuition prevails. The risk of making mistakes is not due to the use of the rapid mode in situations requiring this process. Rather, the risk lies in using it in situations in which the analytical method is necessary or in not using metacognition, a kind of cognition characterized by analytical evaluation of the initially made decision, its steps, and the possible biases.^(9)^

In the current context, many patients with respiratory symptoms arrive at emergency rooms and health clinics having been bombarded by constant media and health authority warnings related to the novel coronavirus pandemic. However, many of these patients do not present with clinical manifestations related to the viral infection but rather exacerbations of preexisting diseases (bronchial asthma, chronic obstructive pulmonary disease (COPD), congestive heart failure, among others). The signs and symptoms of flu-like syndrome or severe acute respiratory syndrome may overlap in their respiratory symptoms, which is an obvious confounding factor. However, these patients have started to be referred directly to the ICU for several reasons. First, the time healthcare teams spend in close proximity to these patients has been reduced due to the understandable fear of contamination by healthcare workers. These teams often do not receive personal protective equipment from health institutions for the care of patients - which may also be unavailable given the pandemic. Second, there is underuse of nebulized inhaled bronchodilators due to the inherent risk of aerosol production and the possibility of environmental contamination,^(10)^ which leads to an increased risk of ventilatory decline in patients with exacerbations, such as those with bronchial asthma or COPD. Third, corticosteroid therapy has been given less often because of the increased mortality of patients with pneumonia caused by the novel coronavirus who used this drug in early studies,^(11,12)^ although the World Health Organization and the Centers for Disease Control and Prevention recommend its use in patients with exacerbated COPD, even when affected by pneumonia caused by the new coronavirus.^(11,12)^ Fourth, the overindication of lung computed tomography (CT) scans for diagnosis of pneumonia caused by the novel coronavirus may further confound medical reasoning.^(13,14)^ CT findings do not appear to be diagnostic of the novel coronavirus because they are similar to those of any other viral disease.^(13)^ Chest CT has excellent sensitivity (~97%) but very poor diagnostic specificity (~25%), but presently we may need to accept a slightly lower sensitivity for detecting this disease in favor of increased diagnostic sensitivity of other common diseases, whose frequency and importance we may be temporarily minimizing. Lastly, the screening performed at the gateway of hospitals by a specialty, Emergency Medicine (unfortunately recognized in Brazil only in 2015), is inefficient. Insufficient knowledge on the part of the doctor leads to poor performance at the bedside,^(15,16)^ which is exacerbated by fatigue, sleep deprivation, and high patient load.^(17,18)^ In addition, for the first time, the doors of ICUs, which are still quiet with regard to overcrowding, are open to any patient who receives the “coronavirus” or “COVID” stamp, offsetting the typically (and now more) overcrowded emergency rooms.

Uncertainty must be added to the above factors. Usually, doctors already have difficulty dealing with and admitting patients due to the uncertainty inherent to any diagnosis or treatment performed.^(19)^ In a pandemic caused by a novel pathogen with a changing clinical-epidemiological scenario, in which populations that are older and have more comorbidities are affected, uncertainty emerges.^(20)^ In this context, it is important to differentiate risk from uncertainty. Risks are known and can be quantified in a reflective decision process or even intuited in a heuristic decision-making process. In uncertainty, not all the consequences of a decision are known.^(21)^

### Cognitive biases influencing the decision-making process in the COVID-19 pandemic

There are several cognitive biases that can influence the clinical judgment of medical care teams during the pandemic. The most important are listed here.

### Heuristic

In heuristic diagnostic reasoning, the probability of a diagnosis is influenced by the ease of remembering possible diagnoses.^(22)^ In newspapers, on social networks, in professional conversations, and in all forms of communication, the memory of the novel coronavirus is continuous, overwhelming, and never-ending.

### Anchoring

This occurs when the doctor anchors the diagnosis on the initial information learned in the diagnostic process.^(2)^ The ease of access to the ICU bed and the precariousness of care conditions in emergency rooms could promote the early diagnosis of pneumonia presumptively caused by the novel coronavirus.

### Underadjustment

This is the failure to revise a diagnosis based on subsequent information.^(23)^ This bias is also related to premature diagnostic closure, when the clinician concludes the case before all information is obtained. The underadjustment bias is often associated with the anchoring bias.

### Gambler’s fallacy

This arises when doctors do not realize that cases are inherently independent (unless there is an outbreak).^(24)^ The name derives from the fact that the gambler, after observing a long consecutive series of “evens” in a draw, justifies that the next draw will produce an “odd,” not appreciating that each draw is, in fact, independent of all others. This fallacy arises because people tend to think that a coin tossing sequence should be representative of a random sequence and the typical random sequence is not consecutive.

### Prior diagnoses

Doctors are also affected by previous diagnoses or hypotheses previously applied to patients.^(24)^ Thus, a layperson’s or a patient’s opinion, or the diagnosis suggested or performed by other doctors, is established by a series of intermediaries. In the context of a pandemic, all patients who can pass through the emergency room without needing an evaluation are already diagnosed with the viral disease, and this apparently facilitates the work of everyone in the department.

### Lack of feedback

The availability of feedback to professionals who make decisions that are often intuitive and who fail to include metacognition in their decision-making process is fundamental. Often, emergency physicians fit this description. Unfortunately, the lack of feedback is a common bias that compromises future heuristic decisions because the brain subconsciously processes the lack of feedback as positive feedback.^(25)^

## CONCLUSIONS

We are living a unique moment in history, something we never imagined experiencing. We hope to have learned from the mistakes and successes of the countries that dealt with the novel coronavirus before us. We must prevent intuitive decision-making and insecurity from leading us to exhaust the available critical-care beds before they are truly necessary, while still recognizing the importance of rapid decision-making in emergency situations. We must keep evaluating patients objectively, following the vast available literature and maintaining the calmness that our profession requires. Perhaps the best way to achieve this goal is to practice metacognition and establish ways to provide regular feedback to professionals engaged in inherently rapid decision-making processes.

## References

[1] Haselton MG, Nettle D, Andrews PW, Buss DM (2005). The evolution of cognitive bias. The handbook of evolutionary psychology.

[2] Phua DH, Tan NC (2013). Cognitive aspect of diagnostic errors. Ann Acad Med Singapore.

[3] Royce CS, Hayes MM, Schwartzstein RM (2019). Teaching critical thinking a case for instruction in cognitive biases to reduce diagnostic errors and improve patient safety. Acad Med.

[4] Norman GR, Monteiro SD, Sherbino J, Ilgen JS, Schmidt HG, Mamede S (2017). The Causes of errors in clinical reasoning cognitive biases, knowledge deficits, and dual process thinking. Acad Med.

[5] Isenman L (2013). Understanding unconscious intelligence and intuition "blink" and beyond. Perspect Biol Med.

[6] Almada LF (2012). Processos implícitos não-conscientes na tomada de decisão a hipótese dos marcadores somáticos. Cienc Cogn.

[7] Gigerenzer G, Brighton H (2009). Homo heuriticus why biased minds make better inferences. Top Cogn Sci.

[8] Sloman SA, Gilovich T, Griffin D, Kahneman D (2002). Two systems of reasoning. Heuristics and biases: The psychology of intuitive judgment.

[9] Qiu L, Su J, Ni Y, Bai Y, Zhang X, Li X (2018). The neural system of metacognition accompanying decision-making in the prefrontal cortex. PLoS Biol.

[10] Wax RS, Christian MD (2020). Practical recommendations for critical care and anesthesiology teams caring for novel coronavirus (2019-nCoV) patients. Can J Anaesth.

[11] Centers for Disease Control and Prevention - CDC Interim Clinical Guidance for Management of Patients with Confirmed Coronavirus Disease (COVID-19) [Internet].

[12] World Health Organization (WHO) (c2020). Novel Coronavirus (2019-nCoV) technical guidance: Patient management [Internet].

[13] Ai T, Yang Z, Hou H, Zhan C, Chen C, Lv W (2020). Correlation of Chest CT and RT-PCR Testing in Coronavirus Disease 2019 (COVID-19) in China: a report of 1014 cases. Radiology.

[14] Li Y, Xia L (2020). Coronavirus Disease 2019 (COVID-19): Role of Chest CT in Diagnosis and Management. AJR Am J Roentgenol..

[15] Norman GR, Eva KW (2010). Diagnostic error and clinical reasoning. Med Educ.

[16] Norcini JJ, Lipner RS, Kimball HR (2002). Certifying examination performance and patient outcomes following acute myocardial infarction. Med Educ.

[17] Croskerry P (2009). Clinical cognition and diagnostic error applications of a dual process model of reasoning. Adv Health Sci Educ Theory Pract.

[18] Patel VL, Cohen T (2008). New perspectives on error in critical care. Curr Opin Crit Care.

[19] Clark TK, Yi Y, Galvan-Garza RC, Bermúdez Rey MC, Merfeld DM (2018). When uncertain, does human self-motion decision-making fully utilize complete information. J Neurophysiol.

[20] Han PK, Zikmund-Fisher BJ, Duarte CW, Knaus M, Black A, Scherer AM (2018). Communication of Scientific Uncertainty about a Novel Pandemic Health Threat Ambiguity Aversion and Its Mechanisms. J Health Commun.

[21] Volz KG, Gigerenzer G (2012). Cognitive processes in decisions under risk are not the same as in decisions under uncertainty. Front Neurosci.

[22] Mamede S, van Gog T, van den Berge K.Rikers RM.van Saase JL.van Guldener C (2010). Effect of availability bias and reflective reasoning on diagnostic accuracy among internal medicine residents. JAMA.

[23] Gallagher EJ (2003). Thinking about thinking. Ann Emerg Med.

[24] Croskerry P (2002). Achieving quality in clinical decision making cognitive strategies and detection of bias. Acad Emerg Med.

[25] Stiegler MP, Ruskin KJ (2012). Decision-making and safety in anesthesiology. Curr Opin Anaesthesiol.

